# Tumor Treating Fields (TTFields), and their concomitant application with FOLFOX, are effective for the treatment of gastric cancer cells

**DOI:** 10.3389/fonc.2026.1575083

**Published:** 2026-03-09

**Authors:** Hila Fishman, Hila M. Ene, Einav Zeevi, Naama Flint-Brodsly, Kerem Wainer-Katsir, Roni Frechtel-Gerzi, Antonia Martinez-Conde, Shany Greenstein, Eyal Dor-On, Mijal Munster, Yaara Porat, Tali Voloshin, Shiri Davidi, Gitit Lavy-Shahaf, Adi Haber, Moshe Giladi, Uri Weinberg, Yoram Palti

**Affiliations:** Novocure Ltd., Haifa, Israel

**Keywords:** 5-fluorouracil, antimitotic effect, DNA damage repair, FOLFOX, gastric cancer, oxaliplatin, Tumor Treating Fields (TTFields)

## Abstract

**Background:**

Tumor Treating Fields (TTFields) are electric fields that exert antimitotic effects and impair DNA damage repair in cancerous cells. Gastric cancer, one of the most common types of cancers worldwide, demonstrates poor long-term survival despite advances in therapeutic options. The goal of the current study was to examine *in vitro* the efficacy and mechanism of action of TTFields for treating gastric cancer, and the potential application of TTFields concomitant with FOLFOX, a standard gastric cancer treatment consisting of the therapeutic agents oxaliplatin and 5-fluorouracil [5-FU].

**Methods:**

Human gastric cancer cell lines, AGS and KATOIII, were treated with TTFields using the inovitro system. To test the efficacy of TTFields (alone or together with oxaliplatin, 5-FU, or FOLFOX), cell count, colony formation, and apoptosis were examined. For mechanistic insight, control and TTFields-treated cells were examined by RNA sequencing. Immunofluorescent staining for phospho-histone H2AX (γH2AX), α-tubulin, phospho-histone 3 (pH3), and double stranded DNA was employed for detection of DNA damage, mitotic spindle defects, chromosome mislocalization, and micronuclei clusters, respectively. Expression of DNA damage repair proteins was measured using Western Blot.

**Results:**

Maximal cell count reduction following application of TTFields at various frequencies was identified at 150 kHz. Treatment also led to reduced colony formation, elevated apoptosis, and substantial changes in transcriptomic expression. TTFields-treated cells demonstrated increased DNA damage, elevated expression of DNA damage-related cyclin-dependent kinase (CDK) inhibitors, and reduced expression of proteins from the Fanconi Anemia-BRCA pathway for DNA repair. Treated cells further presented with abnormal mitotic figures, chromosome mislocalization, and micronuclei clusters, indicative of an antimitotic effect of TTFields. The application of TTFields concomitant with oxaliplatin, 5-FU or FOLFOX was more effective than each treatment alone.

**Conclusions:**

The study shows that TTFields induce an antimitotic effect and impair DNA damage repair in gastric cancer cells, and that concomitant treatment with FOLFOX improves treatment efficacy *in vitro*, potentially due to the accumulation of DNA damage. Overall, TTFields treatment holds potential for treatment of gastric cancer alongside standard chemotherapy.

## 1 Introduction

Gastric cancer is a deadly disease, with low survival rates associated with late-stage diagnosis and chemotherapy resistance (1, 2). There is no gold standard therapy for treatment of gastric cancer, and treatment options are selected based on disease stage, histological characteristics, tumor molecular profile, and physician’s choice (1, 2). Surgery, the only curative approach, is employed for early-stage disease; and chemotherapy may be added pre, post, or peri-operatively. For locally advanced and metastatic disease, chemotherapy doublet or triplet combinations are used, with a fluoropyrimidine and a platinum-based agent typically serving as the preferred backbone (1, 3). Such are the FOLFOX regimen, which consists of 5-fluorouracil (5-FU) and oxaliplatin, with added leucovorin (the latter enhancing the efficacy of 5-FU while potentially reducing this agent’s toxicity), and the XELOX regimen, consisting of oxaliplatin and the 5-FU pro-drug capecitabine (1, 3). Recently, immune checkpoint inhibitors (ICIs), such as nivolumab, have been added to the arsenal for patients with programmed death ligand 1 (PD-L1) positive disease; and human epidermal growth factor receptor 2 (HER2)-targeted therapy, such as trastuzumab, for patients with HER2-positive disease (1, 2). While gastric cancer outcomes have improved with these treatment options, patient prognosis remains poor.

Tumor Treating Fields (TTFields) are low intensity (1–3 V/cm), intermediate frequency (100–500 kHz), electric fields that disrupt cellular processes critical for cancer cell viability and tumor progression, ultimately leading to cancer cell death (4, 5). TTFields exert antimitotic effects on cancer cells: (1) the organization of polar tubulin dimers and septin trimers into filaments is disrupted by TTFields thus preventing proper formation of the mitotic spindle and contractile ring, respectively (4–6); and (2) during telophase a non-uniform electric field is generated due to the hourglass shape of the dividing cell, leading to dielectrophoretic forces that interfere with cytokinesis (4, 5). This antimitotic effect leads to chromosome missegragation, formation of micronuclei, and genotoxic stress (7). In the clinic, TTFields therapy is approved concomitant with the antimitotic agent docetaxel (or ICIs) for treatment of patients with metastatic non-small cell lung cancer (NSCLC) (46), and concomitant with gemcitibine and the antimitotic agent nab-paclitaxel for treatment of patients with unresectable, locally advanced pancreatic adenocarcinoma (16).

TTFields have also been shown to promote accumulation of DNA damage and impair DNA damage repair (DDR) mechanisms, associated with decreased expression of proteins from the Fanconi Anemia (FA)-BRCA pathway (8–10). As such, *in vitro* benefit has been demonstrated for TTFields treatment together with DNA-damaging agents (9–11). In the clinic, TTFields therapy is approved concomitant with the DNA alkylating agent temozolomide for treatment of patients with newly diagnosed glioblastoma (GBM), and concomitant with pemetrexed and the DNA intercalating agents cisplatin or carboplatin for treatment of patients with unresectable pleural mesothelioma (12–15). 

The objective of the following *in vitro* research was to examine TTFields effectiveness in gastric cancer, elucidate the underlying mechanism, and examine the therapeutic potential for concomitant application of TTFields with a standard gastric cancer treatment. As effects on DNA damage repair were found pivotal in the response to TTFields in the examined system, the DNA damaging chemotherapy regimen FOLFOX was selected for co-application with TTFields, demonstrating improved efficacy for the concomitant treatment.

## 2 Materials and methods

### 2.1 Cell culture

Human gastric cell lines AGS (intestinal type) and KATO III (diffuse type) were obtained from the American Tissue Culture Collection (ATCC). Cells were grown in F-12K and IMDM media, respectively, supplemented with 10% (v/v) fetal bovine serum (FBS), 2 mM L-glutamine and penicillin/streptomycin (50 µg/ml) in a 37 °C humidified incubator supplied with 5% CO_2_. Media and supplements were purchased from Biological Industries Ltd.

### 2.2 TTFields efficacy experiments – cell count, apoptosis, colony formation, and overall effect

TTFields were applied to the cells using a dedicated system (inovitro, Novocure Ltd.), as previously described (7, 17). Frequency scans were conducted in the range of 100–400 kHz, by treating the cells with TTFields at intensities demonstrating intermediate magnitude of effect, 1.1 V/cm RMS for AGS cells and 1.7 V/cm RMS for KATO III cells. Cell count was measured following 72 h of treatment. All following experiments were performed at 150 kHz TTFields, as it was identified via the frequency scans to be the optimal TTFields frequency for treatment of gastric cells.

TTFields-chemotherapy dose response experiments were conducted with co-application of TTFields and 0.5 to 40 µM 5-FU (Sigma) or 25 to 800 nM oxaliplatin (Sigma). For experiment with the complete FOLFOX combination, IC25 of each drug was employed: 0.5 µM 5-FU with 50 nM oxaliplatin for AGS cells; 1.5 µM 5-FU with 50 nM oxaliplatin for KATO III cells. 5 µM leucovorin (Sigma) was added in both cases to complete the FOLFOX regimen. At treatment end, supernatant was collected, and cells were washed with PBS and collected by trypsinization. Cells were analyzed for cell count and colony formation, and apoptosis was examined on cells combined with their corresponding supernatant, as previously described (10, 18). The expected values for additive effect between TTFields and FOLFOX were calculated as the product of the observed effects for the individual treatments.

Cell counting was performed using flow cytometer (iCyt EC800, Sony Biotechnology) and expressed as a percentage relative to control (**Supplementary Figure 1** for gating strategy). Apoptosis was detected following FITC-conjugated Annexin V (AnnV) and 7-Aminoactinomycin D (7-AAD) cell staining (BioLegend), performed according to the manufacturer’s instructions (**Supplementary Figure 1** for gating strategy) (10, 18). Caspase-3/7 activity was measured using the Vybrant™ FAM™ Caspase-3/7 Assay Kit (Thermo Fisher Scientific, V35118) following the manufacturer’s protocol. Briefly, cells were incubated with the cell-permeant FLICA™ reagent, which irreversibly binds to activated caspase-3/7. After washing to remove unbound dye, positive cell population was detected by flow cytometry (**Supplementary Figure 1** for gating strategy).

For colony formation assay, treated cells were re-plated into 6-well plates (300 cells/well) and grown with no additional treatment for 7–14 days. Colonies were stained with 0.5% crystal violet, quantified and expressed as percentages relative to control. Overall effect was calculated by multiplying the clonogenicity with the corresponding cell count (10, 18).

### 2.3 RNA-seq analysis

TTFields (150 kHz, 1.7 V/cm RMS) were applied to the cells for 24 or 48 h. Then the cells were washed, trypsinized, and collected by 5 min centrifugation at 300g and 4°C. The resulting pellet was resuspended with TRI reagent and RNA purified as previously described (19). RNA was quantified (Qubit, Thermo Fisher Scientific and TapeStation, Agilent). Libraries were constructed using the PolyA fraction, and cleaned up using Agencourt AMPure XP beads (Beckman Coulter), followed by end repair, UMIs addition, adapter ligation and PCR amplification steps. Library generation and sequencing (Illumina NovoSeq 6000 system, Illumina inc.) were done by the Crown Genomics institute of the Nancy and Stephen Grand Israel National Center for Personalized Medicine, Weizmann Institute of Science.

Fastq files were quality control screened with FastQC (20). UMIs from R2 were converted to the header using UMI_tools (21). Reads were aligned to the human genome gencode human reference GRCh38.p13 with annotation file gencode.v36.annotation.gtf (downloaded from https://www.gencodegenes.org/human/) using STAR-2.7.6a (22). Alignment and FastQC were assessed using multiqc (23). Bam files were indexed using samtools index (24). Files were deduplicated using UMI_tools. Reads per gene were counted with HTseq (25). R and Rstudio were used to create gene/sample matrix and further analysis (26, 27). Differential expression was made using Limma-voom (28). A heatmap was constructed to show unsupervised hierarchical clustering of differentially expressed genes (DEGs) with Bonferroni Hochberg adjusted p-value of <0.05, and principal component analysis of gene expression was performed. Gene set enrichment analysis (GSEA) was executed by the GSEA software on the DEGs pre-ranked according to their t score (29) relative to the MsigDB hallmarks gene set and the Reactome pathways (30). A heatmap was constructed to show common significant pathways with FDR ≤0.01 in all samples, and ≤0.001 in at least 3 samples, that reacted to TTFields in the same direction (up or downregulation) for all samples. Visualizations were made using pheatmap (31) and ggplot2 (32). ggrepel was used to add text to volcano plot (33). Gene annotation was taken from the STRINGdb database using STRING package (34) and from Refseq (35).

### 2.4 TTFields mechanism of action experiments – cell cycle, mitotic spindle integrity, micronuclei formation, DNA damage, and protein expression

TTFields (150 kHz, 1 V/cm RMS) were applied for 48 or 72 h to AGS and KATO III cells, respectively. For cell cycle analysis, cells were washed with PBS, trypsinized, fixed, stained with 7-AAD, and examined by flow cytometer (10). Analysis was done by the FlowJo 10.8 software (**Supplementary Figure 1** for gating strategy).

For spindle structure analysis, cells were fixed, stained with rabbit anti-pH3 (Abcam, ab14955, 1:250) and mouse anti-α-tubulin (Sigma-Aldrich, T5168, 1:500) primary antibodies, followed by Alexa Fluor 488-conjugated anti-rabbit and 647-conjugated anti-mouse secondary antibodies (Jackson Immunoresearch, 711-545–152 and 115-605-003, respectively, 1:500) and 4`,6-diamidino-2-phenylindole (DAPI) (Sigma-Aldrich) staining (7). The latter staining also allowed the detection of micronuclei structures.

For detection of DNA damage, cells were fixed and stained with anti-H2A.X phospho (Ser139) antibody (Cell Signaling, 9718, 1:400), followed by AlexaFluor 488-conjugated secondary antibody (Jackson Immunoresearch, 711-545-152, 1:500) and DAPI (10, 36). All fluorescent images were collected using laser scanning confocal system (LSM 700, Zeiss Gottingen). The mean number of foci per nucleus was assessed using the FIJI software with the BioVoxxel plugin. Cells demonstrating a ring of nuclear staining were considered apoptotic and excluded from the analysis. Alternatively, cells were washed with PBS, trypsinized and fixed using 70% ethanol at -20°C. Cells were washed twice with ice-cold PBS and staining was performed using anti-H2A.X phospho (Ser139) AlexaFluor 488-conjugated antibody (BLG-613406). Cells were acquired using flow cytometry and γH2AX positive cells analyzed using the FlowJo software (**Supplementary Figure 1** for gating strategy).

For determination of protein expression, cell extracts were prepared and subjected to western blot analysis (40 μg protein) as previously described (10, 36), using the primary antibodies: anti-p21 (Santa Cruz, SC-6246, 1:500), anti-p27 (Santa Cruz, SC-56338, 1:500), anti-FANCD2 (Cell Signaling, 16323, 1:1000), anti-FANCJ (Cell Signaling, 4578, 1:1000), anti-BRCA2 (Cell Signaling, 10741, 1:1000), anti-GAPDH (Santa Cruz, SC-32233, 1:2000). Horseradish peroxidase (HRP)-conjugated secondary antibody (Abcam, ab97023 or ab6721, 1:2000) and gel imager (GeneGnome XRQ, AlphMetrix Bitech).

### 2.5 Statistical analysis

All experiments were repeated at least three times, and data are presented as mean ± standard error of the mean (SEM). Statistical significance was calculated using GraphPad Prism 10 software (La Jolla, San Diego, CA, USA), with the specific tests used mentioned in figure legends. Differences were considered significant at values of: *p < 0.05, **p < 0.01, ***p < 0.001, and ****p < 0.0001.

## 3 Results

### 3.1 TTFields demonstrated efficacy and triggered transcriptomic changes

**Figure 1:**
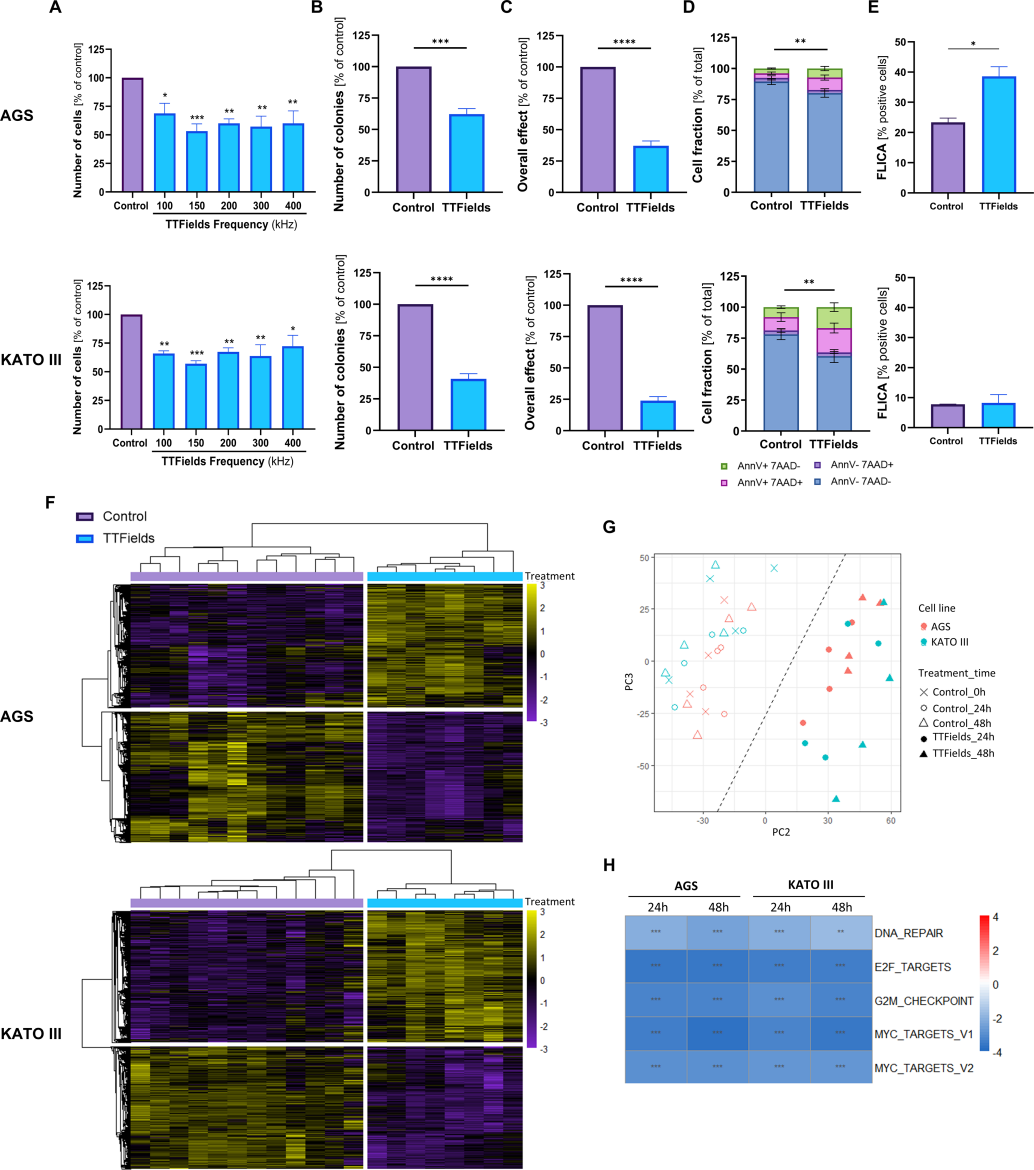
TTFields demonstrated efficacy and triggered transcriptomic changes. **(A-D)** Efficacy examinations following 72 h TTFields application to AGS or KATO III gastric cell lines: **(A)** cell counts at various frequencies; and **(B)** colony formation, **(C)** overall effect, **(D)** apoptosis, and **(E)** active caspase-3/7 levels at 150 kHz TTFields. Values are mean ± SEM. *p < 0.05, **p < 0.01, ***p < 0.001, and ****p < 0.0001 relative to control; One-way ANOVA for **(A)** Student’s t-test for **(B, C, E)** Two-way ANOVA with Sidak’s multiple comparisons for **(D)** showing significance for AnnV^–^/7AAD^–^ fraction. **(F-H)** Transcriptomics analysis following 24 or 48 h treatment with 150 kHz TTFields: **(F)** Heatmap showing unsupervised hierarchical clustering of differentially expressed genes with Bonferroni Hochberg adjusted p-value of <0.05; **(G)** Principal component analysis of gene expression; **(H)** GSEA heatmap of common significant pathways with FDR ≤0.01 in all samples, and ≤0.001 in at least 3 samples, that reacted to TTFields in the same direction (up or downregulation) for all samples.

TTFields frequency scans revealed significantly reduced cell count for AGS and KATO III cells (**Figure 1A**). Maximal cell count decrease for both gastric cell lines was seen at 150 kHz, to a level of 53% for AGS cells and 57% for KATO III cells relative to control; hence, this frequency was utilized in all subsequent experiments. The colony-forming ability of the cells surviving the 72-h exposure to TTFields decreased significantly to 62% and 41% of control for AGS and KATO III cells, respectively (**Figure 1B**). Significant reduction of the overall effect, the outcome of multiplying cell count with the colony-forming ability, was seen for cells treated with TTFields relative to control, to 37% for AGS cells and 24% for KATO III cells (**Figure 1C**). Apoptosis analysis revealed a significant decrease in the live cells fraction (AnnV^–^/7-ADD^–^ stained cells) from 89% for non-treated AGS cells to 80% following application of TTFields, and from 78% for non-treated KATO III cells to 60% after delivery of TTFields, with doubling of the apoptotic population (AnnV^+^/7-ADD^–^ and AnnV^+^/7-ADD^+^ stained cells) (**Figure 1D**). Apoptosis was found to be caspase dependent for AGS and caspase independent for KATO III, as indicative by the FLICA assay results (**Figure 1E**).

In order to elucidate cellular mechanisms participating in the observed efficacy, RNA sequencing was performed on non-treated AGS and KATO III cells (at times 0, 24, and 48 h) and on cells treated with TTFields for 24 or 48 h. The data were examined by unsupervised hierarchical clustering of differential expressed genes (**Figure 1F**) and Principal Component Analysis (PCA, **Figure 1G**). The clear separation between control and TTFields-treated cells indicates differences in the expression profiles. Changes of the most dysregulated DEGs are listed in **Supplementary Table S1** and **S2**, showing no significant overlap between the two examined cells lines. Accordingly, to identify common responses to TTFields, GSEA was performed according to the MSigDB hallmark pathways. This revealed significant downregulation of pathways associated with cell cycle and proliferation, including MYC targets, E2F targets, and G2M checkpoint, as well as of pathways for DNA repair for both cell lines at the two examined time points (**Figure 1H**).

### 3.2 TTFields induced formation of abnormal mitotic figures and micronuclei clusters

**Figure 2:**
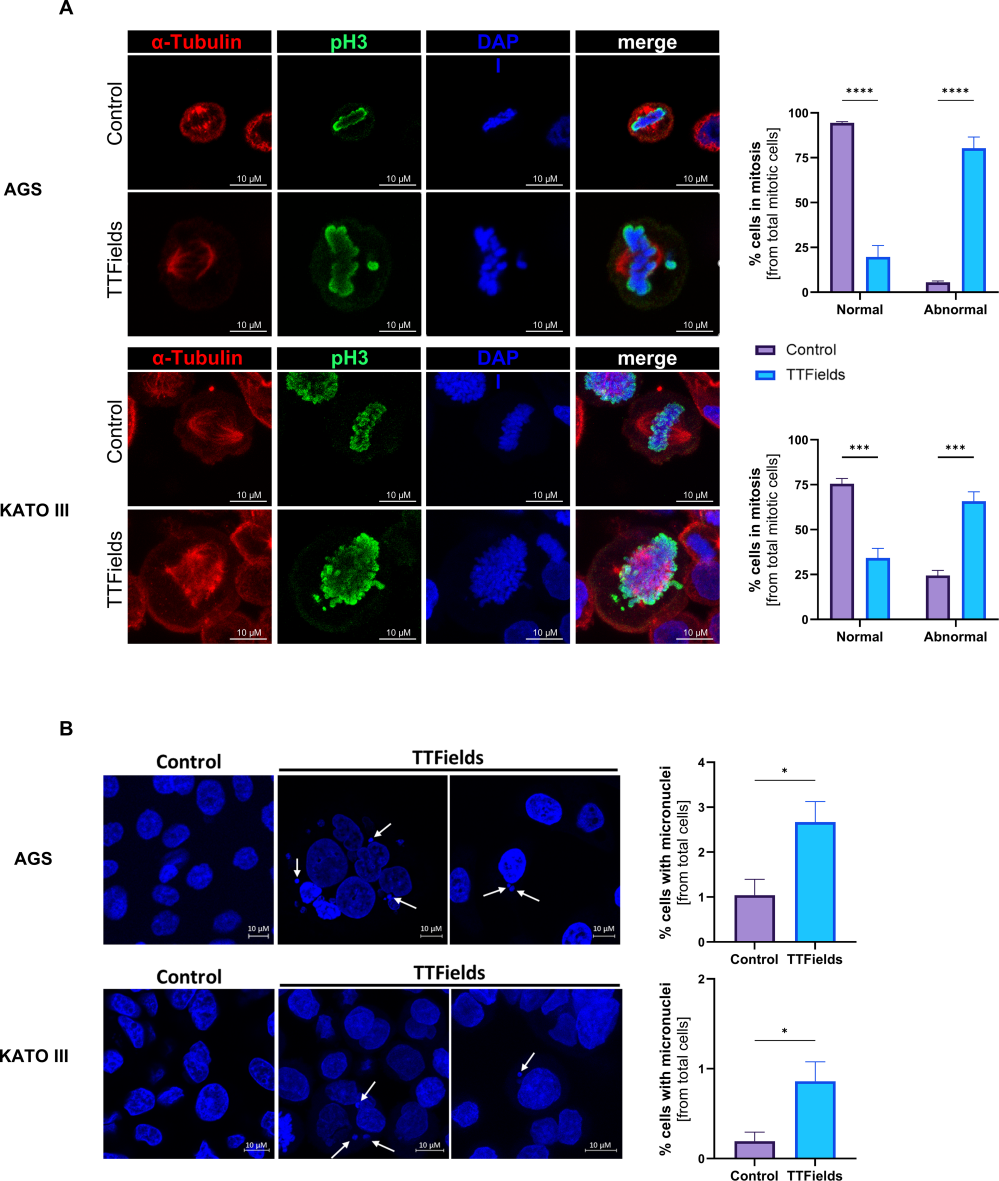
TTFields induced formation of abnormal mitotic figures and micronuclei clusters. Representative images and quantification of abnormal mitotic figures **(A)** and micronuclei clusters **(B)** following 150 kHz TTFields application to AGS and KATO III cells (48 and 72 h, respectively). Blue, DAPI DNA staining; Red, tubulin staining; Green, phospho-histone 3 (pH3) staining, as a marker for chromosome condensation. For B white arrows point to micronuclei clusters. Values are mean ± SEM. *p < 0.05, ***p < 0.001, and ****p < 0.0001 relative to control; Two-way ANOVA with Sidak’s multiple comparison for **(A)** Student’s t-test for **(B)**.

To address the antimitotic effect of TTFields (7), which is manifested in the transcriptomic analysis as downregulation of MYC targets, E2F targets, and G2M checkpoint, confocal fluorescent microscopy with staining for tubulin and DNA (DAPI for chromosomes and anti-pH3 for histones) was performed, revealing elevated levels of abnormal mitotic spindle geometries in cells treated with TTFields (**Figure 2A**). For AGS cells, about 5% of mitotic cells under control conditions displayed abnormal figures, elevating to 80% when TTFields were applied to the cells. In KATO III cells, the percentage of mitotic cells undergoing aberrant mitosis elevated from 25% in control to 65% following TTFields treatment. Increased formation of micronuclei clusters was also seen, which was in both cell lines about 3-fold higher in TTFields-treated versus control cells (**Figure 2B**).

### 3.3 TTFields generated DNA damage and downregulated DNA damage repair pathways

**Figure 3:**
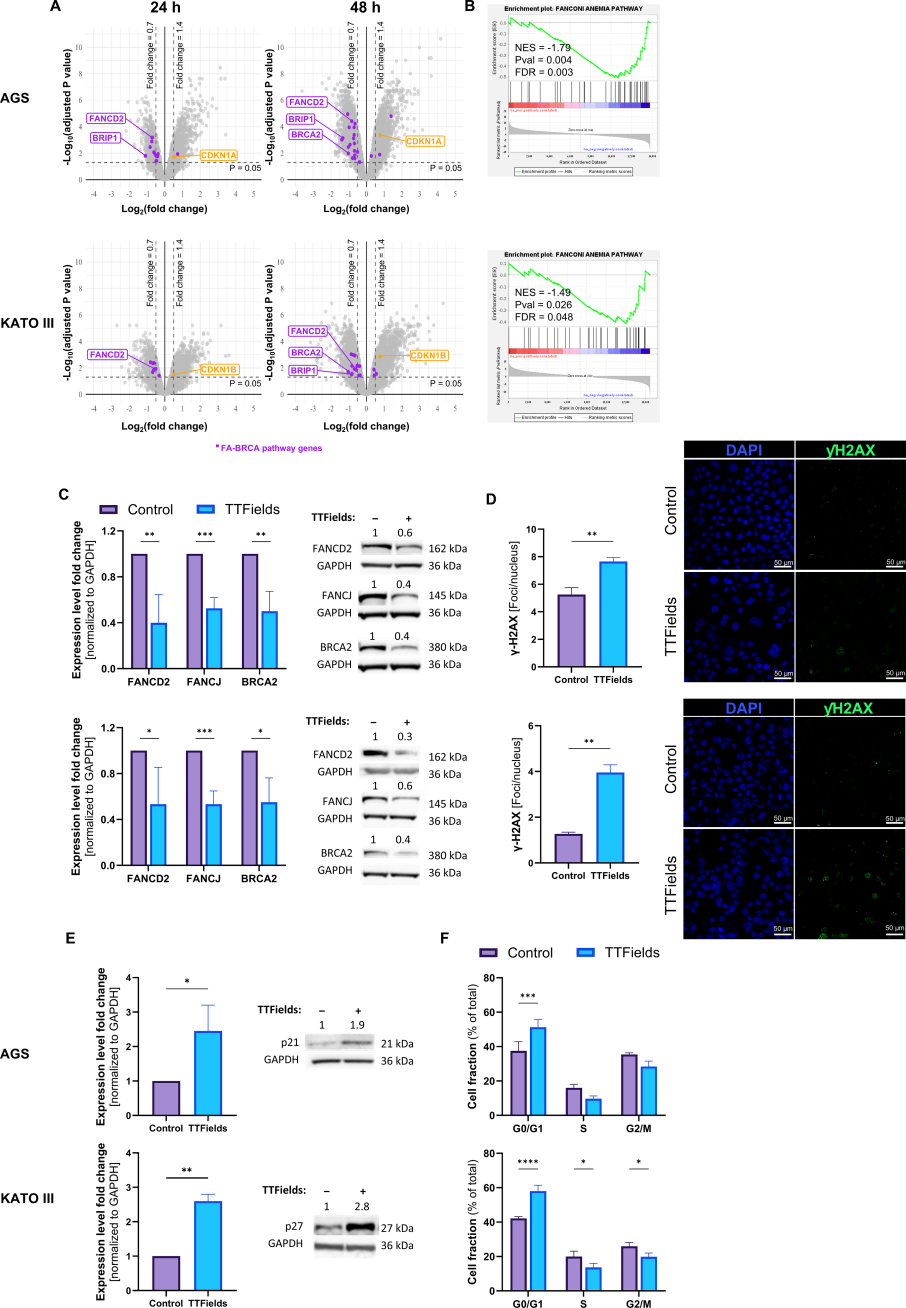
TTFields downregulated DNA damage repair and generated DNA damage. Volcano plots of mRNA expression fold change (150 kHz TTFields versus control, at 24 and 48 h treatment) versus significance. Genes from the FA-BRCA pathway are colored in purple. CDKN1A and CDKN1B are colored in orange **(A)**. GSEA analysis of TTFields compared to control of FA-BRCA pathway at 48h **(B)**. Expression of proteins from the FA-BRCA pathway **(C)**, representative images and quantification of DNA damage (Blue – DAPI, Green – γH2AX) **(D)**, protein expression of CDK inhibitors **(E)**, and distribution of cell cycle phases **(F)** following 150 kHz TTFields application to AGS or KATO III cells (48 and 72 h, respectively). Values are mean ± SEM. *p < 0.05, **p < 0.01, ***p < 0.001, and ****p < 0.0001 relative to control; Student’s t-test for **(D–E)**; Two-way ANOVA with Sidak’s multiple comparison for **(C, F)**.

In accordance with the transcriptomic results, effects on DNA repair were tested. A major DNA repair pathway that has previously been shown to be downregulated following TTFields application is the FA-BRCA pathway (9, 10, 36, 37). Comparison of mRNA expression of genes from this pathway for TTFields-treated cells versus control by volcano plots (**Figure 3A**, purple dots) and GSEA analysis (**Figure 3B**) demonstrated downregulation of the pathway following TTFields application to the gastric cancer cells. Downregulation was evident following 24 h treatments and became even more pronounced after 48 h of treatment. Protein expression levels of representative members of this family were tested, with FANCD2, BRIP1/FANCJ, and BRCA2 all downregulated about 2-fold in both cell lines (**Figure 3C**).

Genotoxic stress following TTFields application was examined by confocal fluorescent microscopy measurements of γH2AX within the nucleus, a surrogate marker of DNA damage. Accumulation of γH2AX following TTFields application was observed in both gastric cancer cell lines (**Figure 3D**). In accordance, expression levels of the cyclin-dependent kinase (CDK) inhibitors involved in DNA damage-induced cell cycle arrest were elevated. mRNA levels of CDKN1A in AGS cells and of CDKN1B in KATO III cells increased following treatment with TTFields for 24 h, and to a higher extent after 48 h of treatment (**Figure 3A**, orange dots). Examination of protein expression level showed an increase of 2- to 3-fold for p21 (CDKN1A) in AGS cells, and of p27 (CDKN1B) in KATO III cells (**Figure 3E**). In accordance with the elevated expression of these CDK inhibitors, cell cycle analysis showed arrest of TTFields-treated cells, with significant elevation of cells in G0/G1 in TTFields-treated versus control cells, from 37 to 51% and from 42 to 58% in AGS and KATO III, respectively, at the expense of the other cell cycle phases (**Figure 3F**). The transcriptomics data further supports the cell cycle arrest results, with downregulation of various cell cycle related genes, including CDK1, CDK2, WEE1, AURKA, and AURKB (**Supplementary Figure 2**).

### 3.4 Concomitant application of TTFields with FOLFOX enhanced treatment efficacy

**Figure 4:**
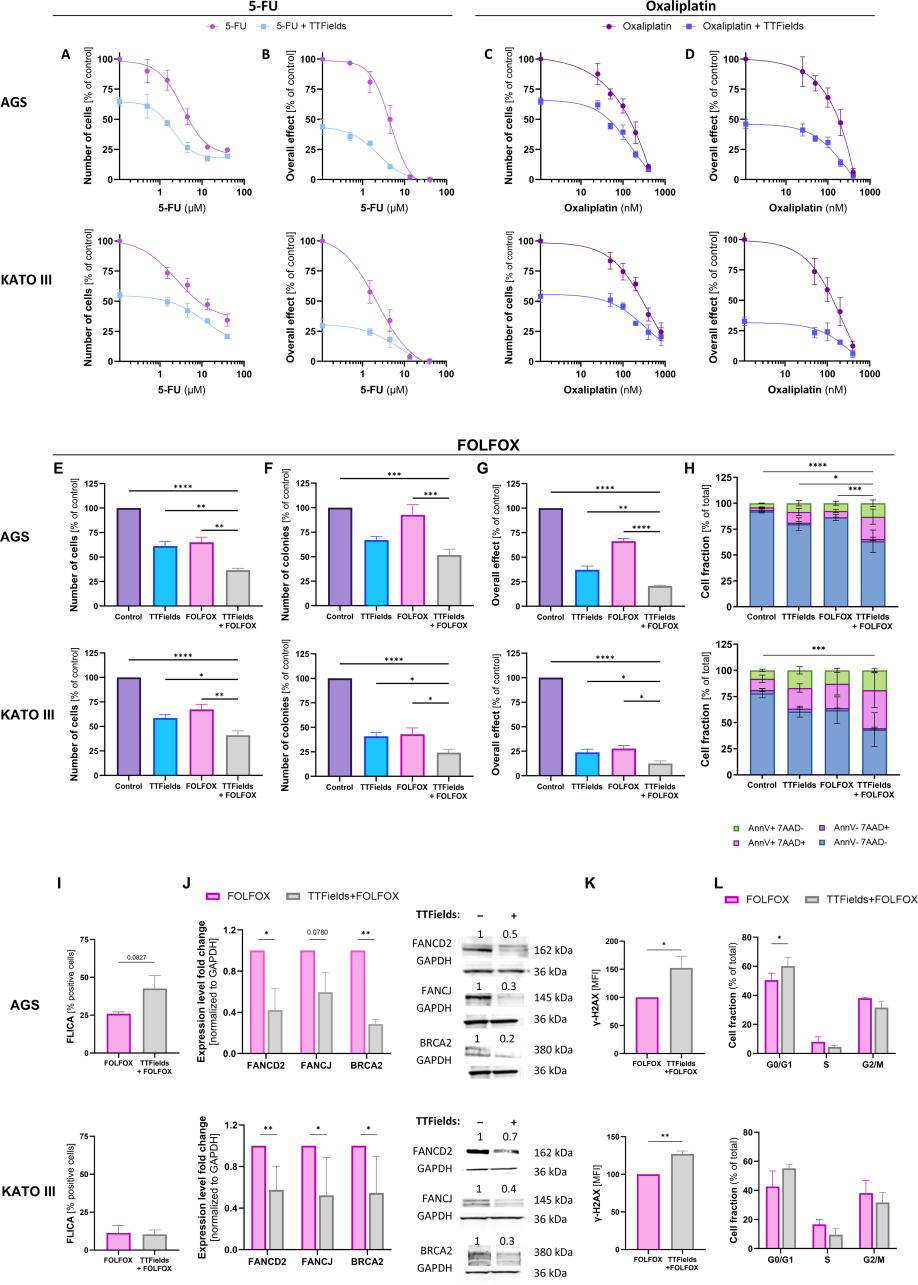
Concomitant application of TTFields with FOLFOX enhanced treatment efficacy. Cell counts **(A, C)** and overall effect **(B, D)** following 72 h application of various doses of 5-FU **(A, B)** or oxaliplatin **(C, D)** to AGS and KATO III cells, with (blue lines) or without (purple lines) 150 kHz TTFields. Values are mean ± SEM. *p* < 0.0001 for dose effect in all cases; *p* < 0.0001 for TTFields effect in all cases; Two-way ANOVA. Cell counts **(E)**, colony formation **(F)**, overall effect **(G)**, and apoptosis analysis **(H)** following 72 h application to AGS and KATO III cells of 150 kHz TTFields, FOLFOX (0.5 µM 5-FU, 50 nM oxaliplatin, and 5 µM leucovorin for AGS cells; 1.5 µM 5-FU, 50 nM oxaliplatin, and 5 µM leucovorin for KATO III cells), or TTFields and FOLFOX together. Values are mean ± SEM. *p < 0.05, **p < 0.01, ***p < 0.001, and ****p < 0.0001 relative to TTFields + FOLFOX; One-way ANOVA with Dunnett’s multiple comparisons for **(E, F)** Two-way ANOVA with Dunnett’s multiple comparisons for **(G)** showing significance for AnnV^–^/7AAD^–^ fraction. Active caspase-3/7 levels **(I)**, expression of proteins from the FA-BRCA pathway **(J)**, quantification of DNA damage **(K)**, and distribution of cell cycle phases **(L)** for TTFields with FOLFOX versus FOLFOX alone. Values are mean ± SEM. *p < 0.05, **p < 0.01, ***p < 0.001, and ****p < 0.0001 relative to FOLFOX; Student’s t-test for **(I, K)**; Two-way ANOVA with Sidak’s multiple comparison for **(J, L)**.

As FOLFOX is part of the standard of care in gastric cancer and contains genotoxic drugs, and since effects on DNA repair have been shown to be pivotal in the response of gastric cancer cells to TTFields, the efficacy of concomitant application of TTFields with FOLFOX was examined by measurements of cell count, colony formation, overall effect, and apoptosis. First, TTFields were co-applied with the two therapeutic components of FOLFOX alone, 5-FU and oxaliplatin. Both gastric cell lines displayed dose-dependent sensitivity to each chemotherapy agent, and the addition of TTFields augmented the effect (**Figures 4A–D**).

Next, the efficacy of TTFields application together with the complete FOLFOX regimen (5-FU, oxaliplatin, and the non-therapeutic component leucovorin) was tested. In AGS cells, cell counts were reduced to 61% and 65% relative to control for TTFields and FOLFOX alone, respectively. Co-application of TTFields and FOLFOX led to an additional reduction in the number of cells to 37% (**Figure 4E**). Colony count was 67% for TTFields alone, practically unaffected by FOLFOX alone (93%), and 52% for TTFields together with FOLFOX (**Figure 4F**). The overall effect was 37% for TTFields alone, 64% for FOLFOX alone, and 21% for co-application of TTFields and FOLFOX, relative to control cells (**Figure 4G**). Apoptosis analysis revealed a decrease in the live cells fraction (AnnV^–^/7-ADD^–^ stained cells) from 92% for non-treated AGS cells to 80% following application of TTFields, to 86% following treatment with FOLFOX, and to 63% after delivery of both TTFields and FOLFOX (**Figure 4H**), in a caspase-dependent manner (**Figure 4I**). Calculations of expected additive effect for TTFields with FOLFOX based on the effects of the individual treatments resulted in 40% for cell count, 62% for colonies, and 25% for overall effect. The calculated values were somewhat higher than the measured values (37%, 52%, and 21%, respectively), hence pointing to an additive effect with slight tendency to synergy.

In KATO III cells, cell counts were reduced relative to control to 57% for TTFields treatment and to 67% for FOLFOX application, while TTFields concomitant with FOLFOX reduced cell number to 41% (**Figure 4E**). Colony count was 41% for TTFields alone, 43% for FOLFOX alone, and 24% for TTFields together with FOLFOX (**Figure 4F**). Overall effects were reduced to 24% and 28% relative to control for treatment with TTFields and FOLFOX alone, respectively. A decrease to 13% was obtained for co-application of TTFields and FOLFOX (**Figure 4G**). The fraction of live cells declined from 78% for non-treated cells to 60% following TTFields application, to 62% following FOLFOX treatment, and to 43% after delivery of both TTFields and FOLFOX (**Figure 4H**), in a caspase-independent manner (**Figure 4I**). Calculations of expected additive effect for TTFields with FOLFOX resulted in 39% for cell count, 18% for colonies, and 7% for overall effect (versus measured 41%, 24%, and 13%, respectively). As the calculated values were somewhat lower than the measured values, an additive effect with slight tendency to antagonistic interaction could be derived for this cell line. Nevertheless, for all measured outcomes in the two examined cell lines, efficacy was higher when TTFields were applied together with FOLFOX as compared to control and to TTFields or FOLFOX alone.

Next, the manifestation of the TTFields mechanism of action when applied together with FOLFOX was examined. As was seen for TTFields alone, the addition of TTFields to FOLFOX treatment downregulated expression of FA-BRCA pathway proteins (**Figure 4J**), elevated levels of DNA damage (**Figure 4K**), and induced cell cycle arrest at G0/G1 (**Figure 4L**) in both examined cell lines, as compared to treatment with FOLFOX alone.

## 4 Discussion

The described research aimed to examine, in cell cultures models, the efficacy and mechanism of action of TTFields for treating gastric cancer, and to examine their potential for enhancing the efficacy of standard gastric cancer therapy. For this purpose, two established gastric cancer models that represent different histological subtypes were selected. Maximal TTFields effectiveness occurs at a specific frequency for different tumor types (4, 5), hence the first step was to perform frequency scans. In both human gastric cell lines, TTFields at 150 kHz led to the greatest cell count reduction, with the AGS cell line demonstrating greater sensitivity to TTFields relative to the KATO III cells. Delivery of TTFields at this frequency also resulted in elevation of apoptosis, indicative of direct cytotoxicity of TTFields. Notably, apoptosis was mediated predominantly through a caspase-dependent pathway in AGS cells, whereas a caspase-independent mechanism was observed in KATO III cells. Such cell line–specific differences in cell death pathways have been reported previously for other treatments (38). The differences may be attributable, at least in part, to the p53 loss-of-function status of KATO III cells (39). Interestingly, previous work in hepatocellular carcinoma models similarly demonstrated higher sensitivity to TTFields in p53 wild-type cells compared with p53-mutant counterparts (18). While these observations suggest that p53 status may influence TTFields responsiveness, additional studies across multiple tumor models will be required to determine the generality of this association and to elucidate the underlying mechanisms.

The increased level of apoptosis, together with the known antimitotic effects of TTFields, suggest that cells may be experiencing abnormal mitosis, pushing them to undergo mitotic cell death (7). Examination of changes in the phenotypes of mitotic cells (which represented about 10% of the total population, data not shown) revealed a significant increase in the fraction of cells undergoing abnormal mitosis when TTFields were applied. Such abnormal mitotic figures may lead to mitotic catastrophe and cell death; however, some cells may complete mitosis to generate daughter cells with abnormal chromosome segregation (7). Indeed, the formation of micronuclei structures in the cells and the lower colony forming ability following TTFields application suggest induction of damage that reduces the ability of progeny cells to further proliferate.

Transcriptomic analysis revealed major changes in gene expression following the application of TTFields, with the most pronounced being downregulation of cell cycle and DNA repair pathways. This was validated on the protein and cellular levels, with demonstrated cell cycle arrest, elevated expression of the CDK inhibitors p21 and p27 (CDKN1A and CDKN1B, respectively) associated with DNA damage induced cell cycle arrest, increased levels of the DNA damage surrogate marker γH2AX, and reduced expression of proteins from the FA-BRCA pathway for DNA repair. The latter finding suggests formation of a cellular state of BRCAness, previously shown in other tumor types (8–10, 36, 37, 40, 41), demonstrating this is a robust pan-cancer mechanism of TTFields. The state of BRCAness induced by TTFields has previously been demonstrated to enhance cellular sensitivity to DNA damaging agents in other tumor types (8, 10, 36, 37).

The FOLFOX regimen for gastric cancer treatment is composed of two DNA damaging agents, 5-FU and oxaliplatin. In contrast to the response to TTFields, sensitivity to FOLFOX was greater in KATO III than in AGS cells. This difference may be attributable, at least in part, to the p53 loss-of-function of the KATO III cells, which has been associated with increased vulnerability to DNA-damaging agents. When TTFields and FOLFOX were applied together, treatment efficacy was enhanced compared with either modality alone in both cell lines; however, the nature of this interaction differed. In AGS cells, the co-application showed a tendency toward synergism, whereas in KATO III cells a tendency toward antagonism was observed. In the present study, the limited additional benefit of adding TTFields to FOLFOX in KATO III cells may reflect their pronounced baseline sensitivity to DNA damage. In this context, the p53 loss-of-function status of KATO III cells may render them highly susceptible to FOLFOX-induced cytotoxicity, thereby reducing the incremental impact of TTFields-mediated downregulation of DNA damage repair pathways. This interpretation is consistent with the observation that, despite the apparent antagonism, concomitant treatment remained more effective than either monotherapy alone. 

Based on the results of this preclinical study, a phase 2 pilot study was conducted testing TTFields therapy together with XELOX (equivalent to FOLFOX, only with an oral 5-FU), with or without trastuzumab, for treatment of patients with unresectable, locally advanced or metastatic gastric cancer, demonstrating tolerability and encouraging clinical outcomes (42).

In patients with good performance status and no comorbidities, the antimitotic agent docetaxel may be added on top of FOLFOX – the FLOT regimen – for achieving higher response rate (43); however, this approach comes with the cost of higher toxicity (1, 3). As TTFields display an antimitotic effect in gastric cancer cells, concomitant TTFields with FOLFOX can potentially mimic the FLOT regimen. Since TTFields are not associated with systemic adverse effects (only mild skin irritation underneath the arrays) (44, 45), the concomitant application of TTFields with FOLFOX may provide the improved effect associated with the FLOT regimen without its accompanied higher toxicity.

Recently, ICIs emerged as a treatment option in the fight against gastric cancer (1, 2). TTFields therapy was approved in concomitance with ICIs (or docetaxel) for treatment of patients with metastatic NSCLC (46), and has shown efficacy in a phase 2 study in newly diagnosed GBM when applied together with pembrolizumab (47). Furthermore, TTFields were shown to activate the cGAS/STING machinery and induce immunogenic cell death in cancer cells, with downstream activation of a systemic immune response (48–50). This suggests future studies should evaluate immunological effects of TTFields in gastric cancer and the concurrent use of TTFields together with ICIs for treatment of gastric cancer.

## 5 Conclusions

To conclude, this study revealed insights on the mechanism of action of TTFields in gastric cancer, consisting of both an antimitotic effect and DNA damage repair downregulation, and demonstrated the benefit of concomitant TTFields with FOLFOX treatment.

## Data Availability

This data can be found in the European Nucleotide Archive (ENA) at EMBL-EBI as part of accession number PRJEB87601, https://www.ebi.ac.uk/ena/browser/view/PRJEB87601.
