# Supplementary material for: Tumor Treating Fields (TTFields), and their concomitant application with FOLFOX, are effective for the treatment of gastric cancer cells
Source: Front Oncol. 2026 Mar 9;16:1575083. doi: 10.3389/fonc.2026.1575083 (PMC13006209; doi:10.3389/fonc.2026.1575083)
Supplement: Supplementary file 3 [file DataSheet1.pdf]

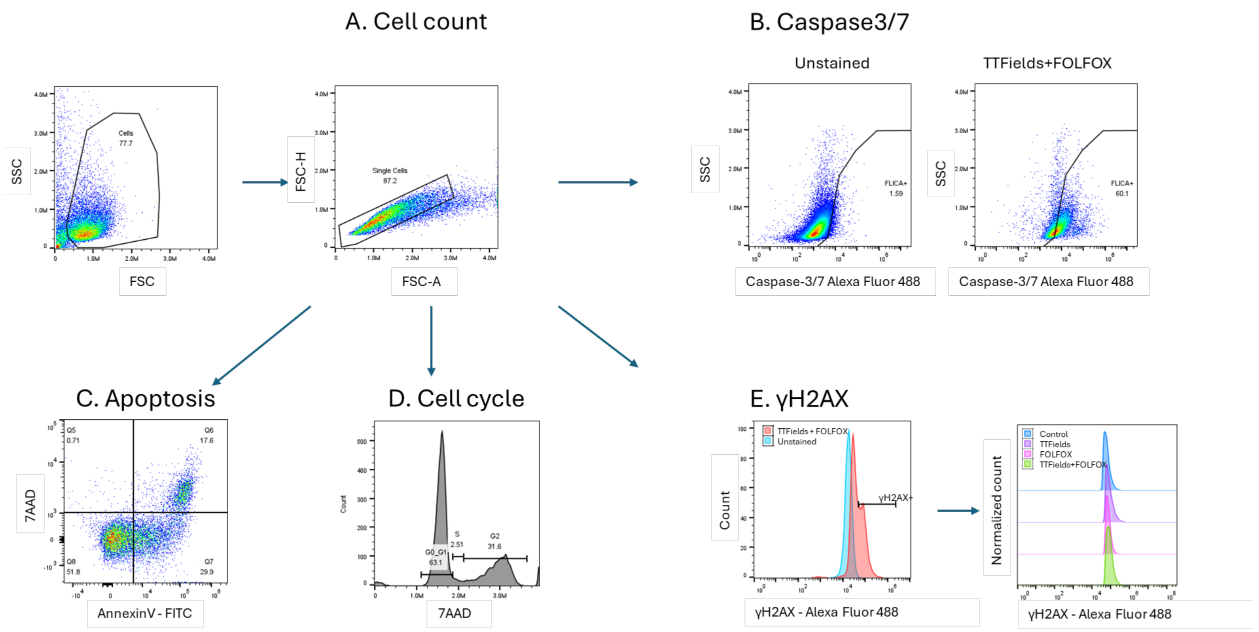

Figure S1: Flow cytometry gating strategy for apoptosis, cell cycle, and DNA damage analysis.

Cells were first gated on FSC vs SSC to exclude debris and select the main cell population. Single cells were then identified using FSC-A vs FSC-H gating. From the singlet population, cell counts were analyzed **(A)**. Caspase-3/7 activity was assessed using FLICA Alexa Fluor 488 staining, with positive populations gated against SSC **(B)**. Apoptosis detection: AnnV–FITC vs 7-AAD plots were used to distinguish live (AnnV^{-}/7-AAD^{-}), early apoptotic (AnnV^{+}/7-AAD^{-}), late apoptotic/necrotic (AnnV^{+}/7-AAD^{+}), and dead (AnnV^{-}/7-AAD^{+}) cells **(C)**. Cell cycle analysis: DNA content was quantified using 7-AAD staining to determine G0/G1, S, and G2/M phase distributions **(D)**. DNA damage detection: γH2AX expression was measured using Alexa Fluor 488 staining. Histograms show γH2AX-positive cells in treatment groups (TTFields, FOLFOX, or combination) compared to unstained and control samples **(E)**.

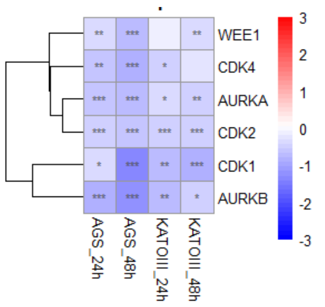

Figure S2: Heat map of transcriptomic changes in cell cycle related genes

Heatmap showing changes in TTFields vs control for the AGS and KATO III cell lines following 24 and 48 h of TTFields exposure. The color corresponds to logFC and the padj values are * ≤ 0.05, ** ≤ 0.01, *** ≤ 0.001
